# Sex Differences in the Prevalence, Outcomes and Management of Hypertension

**DOI:** 10.1007/s11906-022-01183-8

**Published:** 2022-03-07

**Authors:** Paul J. Connelly, Gemma Currie, Christian Delles

**Affiliations:** Institute of Cardiovascular and Medical Sciences, University of Glasgow, 126 University Place, Glasgow, G12 8TA UK

**Keywords:** Sex, Gender, Hypertension, Blood pressure

## Abstract

**Purpose of Review:**

To review recent data on sex differences in the prevalence, outcomes and management of hypertension.

**Recent Findings:**

Although hypertension is overall more common in males, females experience a much sharper incline in blood pressure from the third decade of life and consequently the prevalence of hypertension accelerates comparatively with age. Mechanisms responsible for these blood pressure trajectories may include the sustained vascular influence of hypertensive disorders of pregnancy, interactions between the renin–angiotensin–aldosterone system and sex hormones or even psychosocial gendered factors such as socioeconomic deprivation. Moreover, the impact of hypertension is not uniform and females are at higher risk of developing a multitude of adverse cardiovascular outcomes at lower blood pressure thresholds.

**Summary:**

Blood pressure is a sexually dimorphic trait and although significant differences exist in the prevalence, pathophysiology and outcomes of hypertension in males and females, limited data exist to support sex-specific blood pressure targets.

## Introduction

Hypertension is the leading modifiable risk factor for the development of cardiovascular disease (CVD) and mortality [1]. Blood pressure is a sexually dimorphic trait and prevalence of this condition can vary significantly between males and females across the lifespan [2]. These differences arise from a combination of biological (sex) and psychosocial (gender) mediated factors [3•].

The uniform approach taken in the identification and management of this condition overlooks inherent disparities in prevalence, management and outcomes between males and females. The incremental rise in CVD (i.e. coronary heart disease, ischemic heart disease or myocardial infarction) per 10 mmHg increase in systolic blood pressure is 15% in males, yet is 25% in females [4]. Sex-modulation also occurs across a spectrum of hypertension-mediated conditions such as in heart failure, where females experience a higher disease burden and differing clinical phenotypes (e.g. higher rates of heart failure with preserved ejection fraction), and chronic kidney disease, which is more common in males [5, 6].

**Fig. 1:**
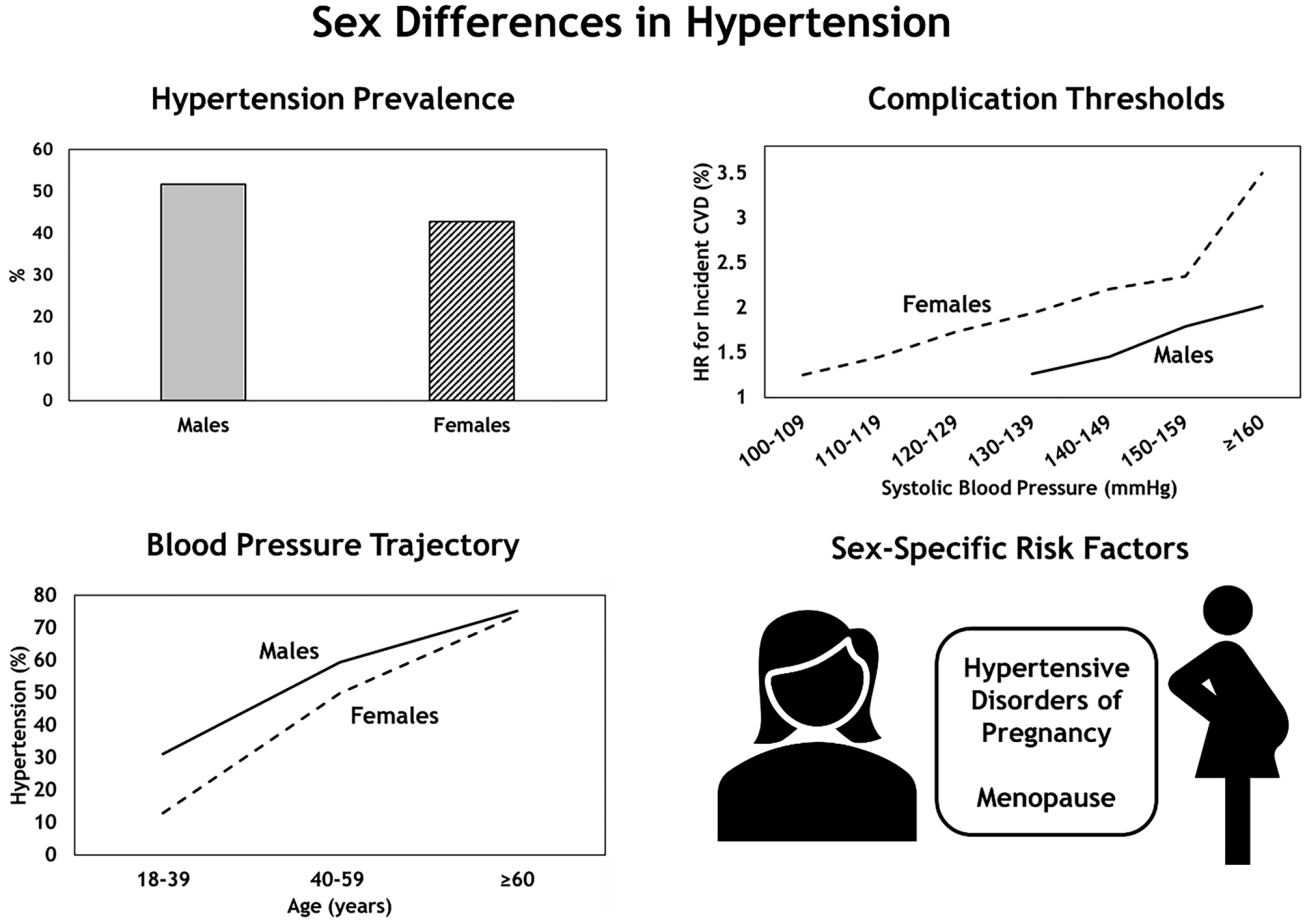
Sex differences in hypertension. There is sexual dimorphism in hypertension prevalence [9], rate of development in hypertension [15] and significant hazard ratios (HR) of incident CVD [40]. Although hypertension remains more common in males, the gradient by which hypertension develops across the lifespan in females is steeper, while the blood pressure thresholds at which CVD develops are lower. These differences may be related to sex-specific risk factors such as hypertensive disorders of pregnancy and the menopause

Despite these fundamental differences (Fig. 1), and with the exception of guidance on the management of hypertension in pregnancy, there are no sex- or gender-specific standards of care in the International Society of Hypertension or American College of Cardiology/American Heart Association guidelines [7, 8]. In this review, we outline recent research relating to hypertension prevalence, mechanisms, outcomes and management with respect to males and females and set forth future research priorities to promote an equitable evidence base.

## Hypertension Prevalence

The prevalence of hypertension between men and women differs greatly. In the Heart Disease and Stroke Statistics 2021 update, the age-adjusted US prevalence of hypertension between 2015 and 2018 in those over the age of 20 years was 51.7% in males and 42.8% in females [9]. The awareness of this condition also differs between sexes. In a nationally representative, cross-sectional study conducted using the Canadian Health Measures Survey between 2007 and 2017 including 5,794,641 people, 23.1% were identified to have hypertension [10]. Importantly, reductions in awareness (~13%), treatment (~17%) and control (~20%) were evident in women over a 10-year period, which were not observed in males. The aetiology of this disparity between males and females is unclear, as no major differences in participant demographics or comorbidities were observed.

These disparities may not be global, as the China Hypertension Survey has demonstrated that awareness (51.9% vs 42.5%), treatment (46.6% vs 35.6%) and control rates (17.7% vs 13.2%) were higher among females compared to males [11]. Additionally, in a cross-sectional study completed in Bangladesh, females demonstrated a higher prevalence and awareness of hypertension compared to males [12]. These differences do highlight the opportunity for sex- and region-specific health interventions to stem the increase of potentially preventable cardiac deaths and disability as a consequence of hypertension.

Gendered factors may also play a significant role in the development of hypertension. Data from 59,805 individuals between the ages of 25 and 69 years from the CONSTANCES cohort in France demonstrated that hypertension prevalence was higher in men than in women [13••]. However, there appeared to be a much stronger relationship between hypertension prevalence and socioeconomic deprivation in women compared to men. Importantly, this was observed in the youngest age group (25 to 34 years) and was particularly associated with education, thereby raising the possibility that early public health measures that tackle socioeconomic inequalities may ameliorate increases in blood pressure in women.

One of the most striking features evident in the hypertension-related differences observed between the sexes is the interaction between blood pressure and age. Until puberty blood pressure is consistent between sexes, where thereafter it is significantly higher in males compared to age-matched females [14]. When stratified by age, males continue to have higher rates of this condition (18–39 years: 31.2 vs 13.0%; 40–59 years: 59.4 vs 49.9%; over 60 years: 75.2 vs 73.9%); however, the rate at which females develop hypertension is much steeper compared to males [15]. Indeed, previous iterations of NHANES data (2013 to 2016) have demonstrated women to have a higher prevalence of hypertension by the sixth decade compared to males [16]. Nevertheless, with increasing age, the females' advantage of protection against hypertension is lost and the development of hypertension accelerates when compared to males.

## Sex and Blood Pressure Trajectories

This upward trajectory of blood pressure in females has been recently demonstrated in a longitudinal blood pressure analysis of 32,833 individuals [17••]. Females in this study exhibited a much sharper incline in blood pressure from the third decade of life. Ultimately, this suggests fundamental differences in drivers for increased blood pressure between the sexes and promotes sex-convergence of hypertension prevalence later in life. Whether these differences are due to sex (e.g. sex hormones, chromosomal complement, pregnancy or epigenetic changes) or gender (e.g. psychosocial traits such as relative economic deprivation) is unclear [18].

A pivotal factor for the accelerated blood pressure trajectory of females may be role of hypertensive disorders in pregnancy. In a study of 58,671 females participating in Nurses’ Health Study II without history of CVD or hypertension at baseline, gestational hypertension and pre-eclampsia during first pregnancy doubled the rate of self-reported chronic hypertension with a mean follow-up of 25–32 years (gestational hypertension HR 2.8 (95% CI 2.6, 3.0); pre-eclampsia HR: 2.2 (95% CI 2.1, 2.3)) [19]. Therefore, transient periods of elevated blood pressure during pregnancy promote a sustained hypertensive phenotype later in life.

The association between gestational hypertension and long-term cardiovascular vulnerability is further supported by data from the Rochester Epidemiology Project medical record-linkage system, based on 9,862 pregnancies between 1976 and 1982 [20•]. In this study, where the respective incidence per woman for hypertensive disorders of pregnancy and pre-eclampsia was 15.3% and 7.5%, the risk of subsequent stroke, coronary artery disease and chronic kidney disease was approximately twice as elevated with a median follow-up of 36.2 years. Taken together, these data highlight the key influence of hypertensive disorders of pregnancy on the lifelong risk of chronic hypertension and its complications.

## Sex Hormones, RAAS and Hypertension

The development of hypertension and the means by which sex modulates the development of this condition is complex and involves numerous systems. Recent advances in our understanding of the relationship between the renin–angiotensin–aldosterone system (RAAS) and sex hormones (oestrogen and testosterone) provide new insight into the sexually dimorphic development of hypertension.

In females, RAAS components, such as plasma renin, fluctuate throughout the menstrual cycle in response to altering levels of estradiol [21]. It has recently been demonstrated that activation of oestrogen receptor α (ERα) and binding to the nuclear oestrogen response element of renin expressing juxtaglomerular cells is necessary for basal renin expression [22]. Therefore, in females the RAAS is influenced significantly by oestrogen status.

Angiotensin (Ang)-(1–7) is a bioactive peptide that acts through the G protein-coupled Mas receptor to oppose the vaso-injurious effects of Ang II and promote vasodilatation, improved endothelial function and inhibits vascular smooth muscle cell proliferation and migration [23]. The vasodilatory action of this peptide differs between males and females, and sex hormones, such as estradiol, may modulate the extent of this response. These effects may be further influenced by age as it has been demonstrated in murine aortic rings that the vasodilatory effect of Ang-(1–7) was lost in older female mice, however, subsequently rescued with estradiol exposure [24]. Moreover, estradiol in this setting reduces levels of reactive oxidative species and improves nitric oxide levels.

It has recently been demonstrated that Ang II-mediated pressor responses are ameliorated via angiotensin type 2 receptor (AT_2_R) activation in female but not male murine models [25, 26]. Importantly, this depressor effect is lost with age and reproductive senescence and restored following oestrogen replacement [27]. This response was associated with the upregulation renal AT_2_R expression, suggesting that the modulation of blood pressure in this setting occurred via a AT2R-mediated renal mechanism. Consequently, both ageing and oestrogen exposure are important mediators of blood pressure modulating effects of RAAS depressor pathways and may be of particular importance in the development of hypertension in post-menopausal women.

Both the role of sex hormone receptors and RAAS mediators in sex differences in hypertension pathophysiology have recently been supported by genomic data. In a study of oestrogen receptor β (ERβ) genetic variants, women with rs10144225 minor alleles were more likely to develop salt-sensitivity of blood pressure [28]. Importantly, this association was only evident in pre-menopausal women who were oestrogen replete, which demonstrated an effect size of 4.4 mmHg per allele. This response may be mediated by an increased aldosterone/renin ratio, which again demonstrates the reciprocity between sex hormones and RAAS mediators. Moreover, in a population of treatment naïve people with hypertension, angiotensin-converting enzyme (ACE)-2 single nucleotide polymorphisms (rs2074192 and rs2106809) were associated with reduced circulating Ang-(1–7) levels in females [29]. These studies help elucidate the pathophysiologic pathways responsible for the susceptibility of some pre-menopausal women to hypertension and facilitate the development of a generalised model of sex differences in blood pressure regulation and hypertension aetiology.

Similarly, interactions between RAAS components and sex hormones are crucial to the development of hypertension in males. It has recently been demonstrated in young male spontaneously hypertensive rats that testosterone supplementation increases blood pressure, which is mediated by the RAAS [30]. However, in ageing rats this supplementation decreases blood pressure by an unknown mechanism, thereby highlighting the importance of both androgen status and age in blood pressure regulation. Importantly, the effect of testosterone-induced blood pressure elevations appears be mediated by Ang-II with an associated increase in the Ang-II receptors (AT1R/AT2R) ratio [31]. The balance between these Ang-II receptor subtypes in the vasculature in response to testosterone may mediate vascular responses to Ang-II and facilitate the development of hypertension in males.

## Sex-specific Hypertension Outcomes

It is well-recognised that risk factors for the development of CVD are not equitable between males and females. In the INTERHEART cohort, where self-reported hypertension was defined as a blood pressure ≥ 140/90 mmHg, blood pressure was a stronger risk factor for myocardial infarction in females compared to males [32]. Recently, in a population-based prospective study from Tromsø, Norway, comprising 33,859 individuals (51% women), it was demonstrated that males experienced an increased risk of myocardial infarction. However, the effect of increasing blood pressure on myocardial infarction risk was more potent in females [33].

This relationship was also demonstrated in a UK Biobank study of 471,998 people (56% female) aged between 40 and 69 years with no CVD at baseline. The incidence of myocardial infarction was 7.76 per 10,000 person years in females and 24.35 per 10,000 person years in males [34]. Although the risk of myocardial infarction was clearly higher in males, the relative risk of myocardial infarction in those with elevated blood pressure was over 80% higher in females. Similarly, the female-to-male ratio of hazard ratios for stage 1 or 2 hypertension was ~ 1.5 and this excess risk did not diminish with age. Moreover, in comparison to normotensive participants, individuals with high blood pressure who were prescribed blood pressure-lowering medication saw elevated hazard ratios for myocardial infarction, which were more marked in females than males (3.65; 95% CI 2.44, 5.44 vs 1.75; 95% CI 1.26, 2.44). Consequently in this cohort, although the incidence of myocardial infarction was lower in females, the impact of hypertension and anti-hypertensive therapy engagement were unfavourable and non-equitable compared to males.

This relationship holds true for younger women also. In a Korean study of 6,424,090 individuals aged 20–39 years, with a median of 13.2-year follow-up, sex-stratified analysis demonstrated that hypertension was associated with higher relative risk of cardiovascular events, including ischemic stroke and myocardial infarction, in women than men [35]. This women-to-men relative risk ratio ranged between 1.14 and 1.46. Comparably, in the Hordaland Health Study of 12,329 participants with stage 1 hypertension (blood pressure 130–139/80–89 mmHg) in their early 40s, hypertension remained a stronger risk factor for myocardial infarction in females compared to males during 16 years of follow-up [36]. In this study, stage 1 hypertension in the 4th decade doubled the risk of myocardial infarction during midlife in females, while this relationship was not observed in males.

The disparate influence of hypertension between the sexes is not limited to ischemic heart disease. In the Campania Salute Network prospective analysis of 4,290 people with treated hypertension and no left ventricular hypertrophy (LVH) at baseline, it was demonstrated that females have twice the risk of developing LVH than males over a median follow-up of 48 months, regardless of the presence of adverse features [37]. Consequently, the attenuation of LVH reversal in response to anti-hypertensive therapy in females compared to males may contribute to higher rates of heart failure, differing disease phenotypes and poorer outcomes in this sex [38]. Moreover, recent data from the prospective REGARDS study has shown that the risk of ischemic stroke with increasing hypertension severity is twice as great in females compared with males, even following adjustment for other conventional stroke risk factors [39].

Similarly, in a pooled analysis of 27,542 participants from established community-based cohort studies, including the Framingham Heart Study, Multi-Ethnic Study of Atherosclerosis, Atherosclerosis Risk in Communities Study and Coronary Artery Risk Development in Young Adults Study, sex-specific thresholds for the development of CVD were observed [40]. The magnitude of risk was consistently demonstrated at lower blood pressure thresholds in females compared to males. This was evident across a variety of CVD including myocardial infarction, heart failure and stroke. These findings call into question whether sex-specific thresholds for the definition of hypertension are required to ameliorate the increased risk associated with elevated blood pressure in females. They also raise the possibility of sex-targeted primary CVD prevention.

## Sex-specific Hypertension Management

### Blood Pressure Targets

Sex-specific thresholds have yet to be included in any major guidelines and evidence regarding their utility from randomised control trials remains controversial. In a sex-specific analysis of the Hypertension Optimal Treatment (HOT) study, reductions in myocardial infarction were mostly observed in women, with a ~ 50% reduction observed in those with a diastolic target of ≤ 90 mmHg compared to targets of ≤ 80–85 mmHg [41, 42]. A 17% decline in myocardial infarction risk was observed in males with diastolic targets of ≤ 80 mmHg compared to ≤ 90 mmHg; however, this was not found to be statistically different. In the Action to Control Cardiovascular Risk in Diabetes (ACCORD) trial, a lack of sex interaction was also observed between the intensive (< 120 mmHg) and standard blood pressure treatment (< 140 mmHg) groups with respect to the primary outcome of first occurrence of a major cardiovascular event in type 2 diabetes [43].

In 2021, the final report for the seminal Systolic Blood Pressure Intervention Trial (SPRINT) was published [44]. This demonstrated that in individuals at increased cardiovascular risk, targeting a systolic blood pressure of less than 120 mmHg resulted in lower rates of major adverse cardiovascular events and all-cause mortality compared to the less intensive target of 140 mmHg endorsed by many clinical guidelines. However, it is not clear whether these impressive results are fully applicable to females as the primary outcome (i.e. first occurrence of myocardial infarction, acute coronary syndrome not resulting in infarction, stroke, acute decompensated heart failure or death from cardiovascular causes) was not found to be statistically significant in females participants (HR 0.84; 95% CI 0.62, 1.14).

The reasons for this are multifaceted. As is evident with so many clinical trials, females were underrepresented in SPRINT, comprising only 35.9% of the intensive and 35.5% of the standard arms, which was much lower than the planned enrolment of 50%. Moreover, the primary outcome occurred less in females than in males in those receiving intensive (4.6% vs 5.5%) or standard (5.4 vs 7.3%) treatment, and therefore, the signal for improved outcomes may not have been as strong in this lower risk population.

In a sex-specific analysis of SPRINT data, Foy et al. demonstrated that in comparison to the standard treatment group, the primary composite outcome in the intensive treatment group was reduced by 16% (95% CI 0.61, 1.13) in women and by 27% in men (95 CI 0.58, 0.89) [45]. As demonstrated by confidence intervals, there was no difference apparent between female treatment groups or interaction between treatment and sex. Consequently, the authors concluded that both sexes experienced comparable effectiveness from intensive blood pressure control.

However, a subsequent SPRINT post hoc sex-specific analysis has been undertaken utilising patient-level data [46]. In SPRINT, randomisation was not stratified according to sex; therefore, propensity score matching was applied to balance baseline characteristics between intensive versus standard treatment groups in both males and females. This analysis demonstrated that males on intensive therapy had a lower risk of the composite outcome compared to those on standard therapy, while no differences in treatment groups were observed in females. This may be explained by the lower baseline cardiovascular risk in females in this analysis.

Ultimately, discerning whether females benefit from intensive blood pressure lowering treatment will not be achieved from post hoc analysis, but the inclusion and engagement with females in clinical trials. Moreover, sex must be factored into trial design with particular focus upon recruitment and outcomes [47]. Lastly, SPRINT was terminated prematurely after ~3 years due to the significant benefit of the intensive strategy. This did not appear to take into account the low recruitment, low event rate and neutral outcome in females. Consequently, clinical research must consider such eventualities to ensure that if trials are discontinued, the benefits or risks of doing so are apparent between sexes.

### Gender Bias and Treatment Inertia

In addition to the fundamental research being undertaken to delineate the correct means and targets for the management of hypertension in males and females, we must also address many factors and biases inherent in everyday clinical practice that preclude optimum therapy and outcomes.

There are numerous studies demonstrating a clear disparity in the prescription of anti-hypertensive therapies between males and females. In the EPIC Norfolk cohort, women were less likely to receive anti-hypertensive therapy than men despite demonstrating comparable blood pressure [48]. A recent meta-analysis assessed gender differences in cardiovascular medication prescription in primary care, including a total of 43 studies and 2,264,600 participants. Of these 28% were women and their ages ranged between 51 and 76 years [49]. The pooled prevalence of anti-hypertensive medication use was 68% in women and 69% in men. Although no significant differences were observed between genders, women were 15% less likely to be prescribed ACE inhibitors and almost 30% more likely to be prescribed diuretics. Similarly, in the Multi-Ethnic Study of Atherosclerosis study of adults aged 45–84 years, after 65 years of age hypertension control rates were lower in females and this treatment-gap widened with increasing age [50]. Consequently, gender biases in relation to the mode and intensity of treatment exist, are potentially enhanced with age and must be addressed in the management of female patients with hypertension.

## Conclusions

Sex differences in blood pressure have been apparent since the 1940s [51], yet our understanding of the mechanisms or clinical relevance of these differences is only now emerging. These recent data demonstrate that the prevalence of hypertension differs between males and females. The trajectory of blood pressure between the sexes is not uniform and may underlie disparate disease processes. The consequences of elevated blood pressure between males and female again appears sex-specific, and there is emerging evidence that hypertension is a more potent cardiovascular risk factor in females.

Despite these clear disparities, we lack sex-specific guidance for the management of our patients, which has the potential to increase health inequalities. These will continue to rise without a concerted effort to engage and raise awareness of hypertension with the general public. We must do more to facilitate the equitable inclusion of female participants in clinical studies to strengthen our evidence base. Lastly, we must address our own biases and swiftly address why so many of our female patients are subject to treatment inertia.
